# TGF-M: Topology-augmented geometric features enhance molecular property prediction

**DOI:** 10.1371/journal.pcbi.1013004

**Published:** 2025-04-22

**Authors:** Wei He, Xu Tian, Xue Li, Peifu Han, Shuang Wang, Lin Liu, Tao Song

**Affiliations:** 1 Qingdao Institute of Software, College of Computer Science and Technology, China University of Petroleum (East China), Shandong Key Laboratory of Intelligent Oil and Gas Industrial Software, Qingdao, China; 2 The Department of Stomatology, The First Medical Center, Chinese PLA General Hospital, Beijing, China; Ocean University of China, CHINA

## Abstract

Accurate prediction of molecular properties is a key component of Artificial Intelligence-driven Drug Design (AIDD). Despite significant progress in improving these predictive models, balancing accuracy with computational complexity remains a challenge. Molecular topological and geometric features provide rich spatial information, crucial for improving prediction accuracy, but their extraction typically increases model complexity. To address this, we propose TGF-M (Topology-augmented Geometric Features for Molecular Property Prediction), a novel predictive model that optimizes feature extraction to enhance information capture and improve model accuracy, and reduces model complexity to lower computational cost. This approach enhances the model’s ability to leverage both topological and geometric features without unnecessary complexity. On the re-segmented PCQM4Mv2 dataset, TGF-M performs remarkably, achieving a low mean absolute error (MAE) of 0.0647 in the HOMO-LUMO gap prediction task with only 6.4M parameters. Compared to two recent state-of-the-art models evaluated within a unified validation framework, TGF-M demonstrates comparable performance with less than one-tenth of the parameters. We conducted an in-depth analysis of TGF-M’s chemical interpretability. The results further validate the method’s effectiveness in leveraging complex molecular topology and geometry during model learning, underscoring its potential and advantages. The trained models and source code of TGF-M are publicly available at https://github.com/TiAW-Go/TGF-M.

## Author summary

Predicting molecular properties is a cornerstone of drug discovery, directly influencing the development of new medicines. Current approaches often rely heavily on computationally expensive 3D structural data, posing challenges for large-scale or real-time applications. In the context of molecular modeling, topology represents the atom-to-atom connectivity within a molecule, while geometry describes the precise spatial arrangement of these atoms. Combining these two aspects allows for a more comprehensive understanding of molecular properties, as topology captures structural relationships and geometry encodes spatial interactions. This work introduces a novel method that combines molecular geometric and topological features to enhance prediction accuracy while significantly reducing computational complexity. By bridging the gap between molecular connectivity (2D topology) and spatial arrangements (3D geometry), our approach not only offers a more efficient pathway to understanding molecular behavior but also demonstrates the potential to make advanced predictive models more accessible. This work paves the way for scalable and interpretable molecular modeling, addressing key challenges in data-driven biology and providing new tools for applications in drug design.

## Introduction

The properties of drug molecules include their physical, chemical, biological and pharmacological characteristics [1]. The HOMO-LUMO gap describes the energy difference between the highest occupied molecular orbital (HOMO) and the lowest unoccupied molecular orbital (LUMO) in a molecule [2]. The size of this gap significantly influences the chemical and physical properties of a molecule and is crucial to understanding and predicting its optical, electrochemical, and chemical reactivity [3]. By understanding and predicting drug molecules’ properties, scientists can optimize molecular structures during drug design, thus enhancing therapeutic efficacy, minimizing side effects, and speeding up the drug development process [4]. Traditional methods for predicting molecular properties, such as the force field method [5] and density functional theory (DFT) [6], have achieved significant success. However, these methods are computationally expensive, often taking several hours to calculate the properties of a single molecule [7], making them impractical for screening large sets of potential drug candidates.

With the advancement of artificial intelligence (AI) [8], molecular property prediction (MPP) have entered a new stage of development [9–11]. By applying advanced AI models, scientists can predict the various properties of potential drug molecules in a short time, significantly improving the efficiency and success rate of drug design. To achieve computer-aided molecular property prediction, the first issue to address is molecular representation, which typically takes three different forms: one-dimensional SMILES sequences [12–14], two-dimensional structural diagrams [15–17], and three-dimensional collections of atoms in space [18–20]. 2D and 3D molecular representations are particularly favored by scientists as they contain rich attributes and potential features that are crucial factors influencing molecular properties. 2D molecular graphs represent atoms as nodes in a heterograph and bonds as edges, providing rich topological information that aids in rapid screening and preliminary prediction, showcasing efficiency advantages, but the accuracy is suboptimal [21]. The relative positional information of atoms in 3D space critically influences quantum mechanical properties as well as other attributes (such as solubility and interactions with proteins) [22]. The detailed spatial conformational information provided by 3D configurations facilitates in-depth analysis of molecular interactions and reactivity but also incurs high computational costs [23–25]. Therefore, considering both accuracy and costs, previous researchers have conducted extensive work, designing models to explore the performance of these two molecular representations in MPP tasks.

Traditional Graph Neural Networks (GNNs), such as Graph Convolutional Networks (GCN) [26] and Graph Isomorphism Networks (GIN) [27], were initially designed to process 2D molecular graphs. However, these models struggled to capture global features. To address this limitation, GCN-VIRTUAL [28] and GIN-VIRTUAL [29] introduced virtual nodes, improving model performance by incorporating global information. CoAtGIN [30] further advanced these models by effectively combining both local and global features. Although these approaches were low computational complexity, their performance on complex tasks remained suboptimal. In response to these limitations, the development of pure Graph Transformers (GTs) [31–33] brought significant improvements in molecular graph learning. For example, the EGT [34] introduced specialized edge channels and cross-layer updates to enhance pairwise representations, improving both node and edge predictions. However, these models were still constrained by their dependence on 2D topology information. Recent works have incorporated 3D geometric information into Transformer architectures, such as GPS++ [35] and Transformer-M [36], which integrate both 2D and 3D features to improve model performance. AEGNN-M [37] further advances this line of work by combining both 2D molecular graphs and 3D spatial coordinate information, improving the model’s ability to capture complex molecular features and enhance prediction accuracy. Uni-Mol+ [38] proposed an efficient approach to generate initial 3D conformations and iteratively refine them to predict quantum chemical properties using a dual-stream Transformer model. TGT [39], on the other hand, bypassed the need for initial 3D coordinates by directly predicting interatomic distances from 2D graphs through a triplet attention mechanism. While incorporating 3D information into Transformer models has significantly improved task performance, it has also increased model complexity and computational demands, leaving room for further optimization.

Based on the above work, we emphasize efficient molecular representation and model design with low computational complexity, and propose a novel method, TGF-M, which effectively balances model prediction accuracy and computational efficiency. Computational complexity, in this context, refers to both space and time complexity. Space complexity is measured by the number of model parameters, while time complexity is quantified using the asymptotic notation *O*. TGF-M introduces an innovative molecular structure feature encoder that specifically combine geometric distances, topological connectivity, and topological degree information, and enhance the representation of geometrical features through topological information, thus achieving efficient molecular feature encoding. Therefore, downstream prediction tasks only require a lightweight predictor to achieve accurate property predictions. The high-performance feature encoding ensures high accuracy, while the lightweight predictor effectively reduces computational complexity, thereby balancing accuracy and computational complexity.

Additionally, we observed the complementary relationship between molecular geometric and topological information and designed specific experiments to deeply explore the enhancement effect of different types of topological information on geometric information.

Building on these contributions, TGF-M demonstrated outstanding performance on the re-segmented PCQM4Mv2 dataset, achieving a record-low MAE of 0.0647 with only 6.4M parameters. The re-segmentation followed official guidelines to ensure consistency between our 3D model and the official dataset, as the original dataset only provides 3D structural information for the molecules in the training set. To further validate the performance of TGF-M, we conducted additional comparative experiments, comparing it with the latest large-parameter models within a unified validation interval.

## Materials and methods

### Problem formulation

The goal of MPP is to correlate the biochemical properties of target molecules. At the heart of this task is molecular representation learning. This process encompasses the encoding of molecules through specific methodologies. It aims to produce accurate representations that facilitate subsequent property prediction tasks. Formally, we can define the MPP problem as equation (1).


$$\begin{array}{ccc} y=F(\text{Encoder}(X)) & & \end{array}$$ (1)

where *y* represents the target property, *X* is the molecular encoding input, *Encoder* is the representation learning model, and *F* is the predictor used for MPP, corresponding to the Feature Engineering and Predictor components in our model.

### Dataset

The large-scale molecular dataset PCQM4Mv2 from the Open Graph Benchmark (OGB) was utilized for training in this study [40]. PCQM4Mv2 is a quantum chemistry dataset originally curated by the PubChemQC project and contains approximately 3.37 million training samples. The dataset includes SMILES strings for the molecules and provides the equilibrium 3D structures exclusively for the training set in SDF format.

Molecular structures can be represented as graphs, where nodes correspond to atoms and edges denote chemical bonds. Each node is associated with a 9-dimensional feature vector, and each edge carries a 3-dimensional feature vector. The atomic spatial coordinates of the 3D molecular structures can be obtained from the SDF format data to derive relevant feature information.

The HOMO-LUMO gap data is used as the prediction target. This data is calculated using DFT. The HOMO-LUMO gap is one of the most important quantum chemical properties of a molecule because it is closely related to reactivity, photoexcitation, and charge transfer.

### Data preprocessing

During the training process, TGF-M requires bond distance information, while the original SDF data typically includes only the relative positional coordinates of atoms. Directly processing these coordinates can lead to excessive computational overhead. Therefore, we proposed a specialized data preprocessing framework to precompute and store geometric and topological information, thereby optimizing model training efficiency.

We calculated the Euclidean distance between each pair of atoms from their positional coordinates to extract spatial geometric information. Additionally, we performed feature extraction on the chemical bonds in 2D molecular graphs, focusing on bond types, stereochemistry, and conjugation, extracting 11 key topological features to explore the complementary relationship between molecular geometric and topological information. The preprocessing algorithm steps are detailed in Algorithm 1 and Algorithm 2.

**Algorithm 1: Pseudocode of geometric extraction.**

**Parameters:***θ* = { *P* = Molecular positions , *EI* = Edge indices , *SM* = Slices for molecules , *SE* = Slices for edges } , *NM* = Number of molecules, *D* = Edge distances, *SD* = Slices for edge distances

**Inputs:**   Data from the original dataset *θ*

**Outputs:**   Target geometry information and its slices *D*, *SD*

1:   **function** Compute-Edge-Distances($P_{m},EI_{m}$)

2:    $P_{s}⇐P_{m}[EI_{m}[0]]$

3:    $P_{e}⇐P_{m}[EI_{m}[1]]$

4:    **return** $\sqrt{\sum {\left(P_{s}-P_{e}\right)}^{2}}$

5:   **end** **function**

6:   *D* ⇐ [ ] , *SD* ⇐ *SE*

7:   **for** *i* ⇐ 0 **to** *NM*–2 **do**

8:    $P_{m}⇐P[SM[i]:SM[i+1]]$

9:    $EI_{m}⇐EI[:,SE[i]:SE[i+1]]$

10:    $D_{m}⇐$ Compute-Edge-Distances($P_{m},EI_{m}$)

11:    append(*D*, $D_{m}$)

12:   **end** **for**

13:   **return** *D*, *SD*

**Algorithm 2: Pseudocode of topology extraction.**

**Parameters:***Ω* = { *EA* = Edge attributes , *SE* = Slices of edges } , *NE* = Number of edges, *D* = Dictionary for edge types, *IT* = Initial type identifier, *T* = Edge types, *ST* = Slices for edge types

**Inputs:**   Data from the original dataset *Ω*

**Outputs:**   Target topological information and its slices *T*, *ST*

1:   **function** Compute-Edge-Type($EA_{i},D,IT$)

2:    $type\text{\_}str{⇐}^{\prime }{-}^{\prime }.\text{join}(\text{map}(\text{str},EA_{i}.\text{tolist}()))$

3:    **if** *type*_*str* ∉ *D* **then**

4:    *D* [ *type*_*str* ] ⇐ *IT*

5:    *IT* ⇐ *IT* + 1

6:    **end** **if**

7:    **return** *D* [ *type*_*str* ] , *D* , *IT*

8:   **end** **function**

9:   *T* ⇐ [ ] , *ST* ⇐ *SE*, *D* ⇐ { } , *IT* ⇐ 0

10:   **for** *i* ⇐ 0 **to** *NE*–1 **do**

11:    $EA_{i}⇐EA[i]$

12:    *type*_*id* , *D* , *IT* ⇐  Compute-Edge-Type($EA_{i},D,IT$)

13:    append(*T*, *type*_*id*)

14:   **end** **for**

15:   **return** *T*, *ST*

Overall, by converting raw 2D/3D molecular data into reusable geometric and topological feature sets, these precomputation steps significantly reduce on-the-fly calculations during training. This not only accelerates the model’s learning process but also enhances reproducibility and extensibility, making it easier to integrate TGF-M into large-scale molecular modeling pipelines.

Given that the OGB official dataset only provides equilibrium 3D molecular structures for the training set, we re-segmented the original training set data following the OGB official dataset partitioning principles to better adapt to the TGF-M model. We conducted model training and validation on this re-segmented dataset, ensuring consistency in data distribution and reproducibility of experimental results.

### The overall framework of TGF-M

**Fig 1:**
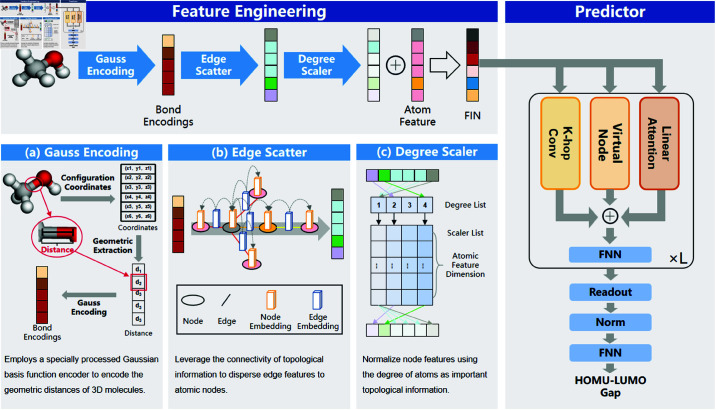
The overarching architecture of the TGF-M, which is divided into two main components: feature engineering and predictor.

As shown in Fig 1, the overall architecture of TGF-M is divided into two main components: feature engineering and predictor. Unlike AEGNN-M [37], which combines 2D molecular graph representations with 3D spatial coordinates through a dual GAT and EGNN framework, TGF-M integrates topology-augmented geometric features within a single, unified feature engineering step. This allows TGF-M to effectively process both 2D topological and 3D geometric information together, achieving an efficient and lightweight architecture. To comprehensively capture the characteristics of molecular graphs, TGF-M introduces a Gaussian encoding method in the feature engineering section to process geometric distance information, generating the initial edge feature encoding. These edge feature encodings are then combined with atomic features through topology-enhanced feature transformations, resulting in the final representation of learning information. This approach ensures that valuable information can be extracted and exploited from molecules of different modalities. In the predictor section, to reduce the computational complexity of the model, TGF-M, inspired by CoAtGIN [30], adopts a lightweight predictor architecture that includes three main components: K-hop convolution, virtual nodes, and linear attention. Through nested loops, this architecture fully utilizes the features encoded in the feature engineering stage, ultimately enabling the prediction of the molecular HOMO-LUMO energy gap. The HOMO-LUMO gap is critical for understanding a molecule’s reactivity and electronic properties, which are essential for optimizing drug design and accelerating the development process. In this section, we will provide a detailed analysis of the components and functions of the TGF-M framework, elaborating on its working mechanisms and advantages.

#### Feature engineering.

In recent years, an increasing number of studies have shown that 2D and 3D molecular representations play a crucial role in molecular graph property prediction, particularly with the significant impact of the relative positions of atoms in 3D space [9,36,39]. Therefore, in the feature engineering module of TGF-M, we comprehensively considered the physicochemical features contained in both 2D and 3D representations of molecules, aiming to develop an optimized method to integrate these two data representations, thereby enhancing the performance of downstream prediction tasks. The following are the specific details of the TGF-M feature engineering.

**Gauss encoding.** As shown in Fig 1A, TGF-M employs a specially processed Gaussian basis function encoder to encode the geometric distances of 3D molecules. The Gaussian basis functions, with their smooth and continuous characteristics, effectively capture subtle variations in these distances [41]. Additionally, these functions can map distances into high-dimensional space, allowing even simple linear models to capture complex molecular interactions. For molecular graph data, Gaussian encoding is advantageous because it can represent both local and global structural information. This sensitivity to atomic position variations enhances the model’s ability to capture intricate geometric relationships, leading to more accurate predictions and better generalization across diverse molecular structures. The specific method for Gaussian encoding is defined as follows:

It extracts geometric distance information from the 3D molecular structure as input.


$$\begin{array}{ccc} \begin{array}{ccc} d & =\sqrt{{\left(x_{1}-x_{2}\right)}^{2}+{\left(y_{1}-y_{2}\right)}^{2}+{\left(z_{1}-z_{2}\right)}^{2}}, & \\ D & =\{d_{i};i=1,2,3,\ldots \;\} \end{array} & & \end{array}$$ (2)

The geometric distances are then encoded using multidimensional Gaussian basis functions, transforming them into high-dimensional feature representations.


$$\begin{array}{ccc} \phi_{e}=\frac{{\text{e}}^{-\frac{1}{2}{\left(\frac{D-u_{i}}{\sigma_{i}}\right)}^{2}}}{\sqrt{2\pi }},\;\Phi =\{\phi_{e};e=1,2,3,\ldots \;\} & & \end{array}$$ (3)

where *Φ* represents the set of high-dimensional feature representations obtained by encoding *D* with Gaussian basis functions. For each dimension, we create distinct, learnable Gaussian parameters $u_{i}$ and $\sigma_{i}$ to ensure diversity in feature representation learning. $u_{i}$ and $\sigma_{i}$ are randomly initialized and learn as the training process changes.

Specifically, for each dimension, we create distinct, learnable Gaussian parameters $u_{i}$ and $\sigma_{i}$, ensuring diversity in feature representation learning. These learnable parameters enable the model to capture complex molecular interactions more effectively.

**Edge scatter.** We leverage the connectivity of topological information to disperse edge features to atomic nodes through the Edge Scatter module. This approach simplifies graph structure processing by reducing it to a graph containing only node features, which decreases the model’s processing complexity while preserving the expressive power of node features and facilitating information transmission within the molecule. This significantly enhances the molecular representation learning capability. As shown in Fig 1B, circles and black lines represent nodes and edges, respectively, while yellow and blue cubes represent node and edge features. Arrows indicate the direction of information flow. The Edge Scatter module establishes connections from edges to nodes, allowing the propagation of information between nodes and edges, thus transferring edge features to nodes. The specific transfer method is defined as follows:


$$\begin{array}{ccc} \psi_{v}=\frac{\underset{u\in N(e)}{\sum }\phi_{u}}{\text{Sum}(N(e))},\;\Psi =\{\psi_{v};v=1,2,3,\ldots \;\} & & \end{array}$$ (4)

where *N* ( *e* )  represents the set of edges connected to node *i*; $\phi_{u}$ represents the feature representation of edge *u*; *Ψ* represents the initial feature representation of node which is obtained by aggregating the features of its connected edges.

**Degree scaler.** Since the degree of an atom is a crucial piece of topological information, reflecting the structural environment of the atom within a molecule [42], TGF-M normalizes node features based on atomic degree information. This normalization ensures that the model can more equitably aggregate information from nodes with different degrees, balancing the impact of each node during feature updates and further enhancing the model’s stability and generalization ability. As shown in Fig 1C, TGF-M independently created a learnable Scaler List to store normalization factors for different degrees and dimensions, which are used to process node features formed after the dispersion of distance-encoded features, enhancing the model’s recognition of Gaussian-encoded information. This process is defined as follows:


$$\begin{array}{ccc} \Psi^{\prime }=\lambda^{\left(\text{d}\right)}\times \Psi & & \end{array}$$ (5)

where $\lambda^{\left(d\right)}$ represents a learnable scaling matrix with dimensions *N*×4 (with *N* being the atomic feature dimension and 4 corresponding to the different atomic degrees). This matrix adjusts the normalization factors for each node based on its degree *d*, and $\Psi^{\prime }$ is the node feature obtained after applying this degree-based scaling.

Finally, the node features obtained through the above steps are combined with the atomic encoding features to generate the final representation learning information FIN.


$$\begin{array}{ccc} F=A+\Psi^{\prime } & & \end{array}$$ (6)

where $\Psi^{\prime }$ represents the features obtained after processing the topology-enhanced geometric information; *A* represents the initial atomic encoding features; and *F* represents the final feature input.

#### Predictor.

**Fig 2:**
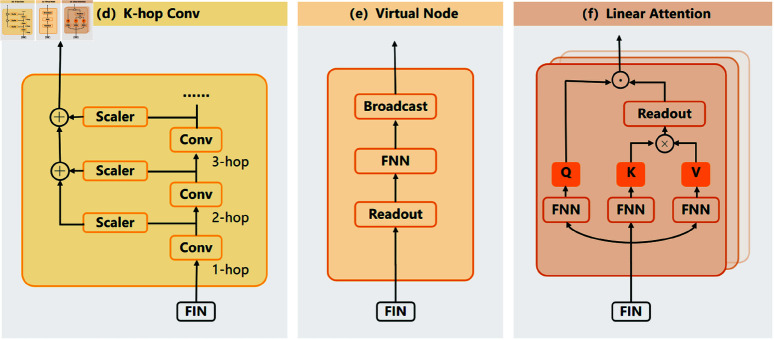
Predictor details. TGF-M adopts a prediction architecture that comprises three key components: K-hop convolution, virtual nodes, and linear attention.

Considering both the model’s complexity and its ability to fully utilize input features, TGF-M adopts a prediction architecture that comprises three key components: K-hop convolution, virtual nodes, and linear attention. K-hop convolution efficiently expands the receptive field to capture both local and long-range interactions. Virtual nodes serve as global aggregators, enhancing communication among distant nodes. Linear attention dynamically weighs feature importance across the graph while minimizing computational overhead. Together, these modules synergistically boost prediction accuracy with minimal complexity. The specific details are shown in Fig 2, and the following sections will provide a detailed introduction to each component.

**K-hop Conv.** The message passing mechanism in graph neural networks relies on feature exchange between nodes. However, single-step feature exchange can only cover information related to chemical bonds near the atoms, failing to capture information such as bond angles or dihedral angles in real molecules, which play a more critical role in MPP tasks [43]. Therefore, to expand the receptive field and capture more complex structural patterns, TGF-M adopts a K-hop convolution strategy, as shown in Fig 2D, aiming to enhance the model’s ability to capture long-range neighbor information within molecules, thereby enabling a deeper understanding of complex molecular structures.

The K-hop convolution strategy can be expressed as follows:

The node feature update rule in traditional graph neural networks is defined as follows. It combines the features of a node with those of its neighboring nodes and utilizes a multilayer perceptron (MLP) to perform a nonlinear transformation on the aggregated features, thereby updating the node’s features:


$$\begin{array}{ccc} h_{v}^{\left(l\right)}={\text{MLP}}^{\left(l\right)}\left(\left(1+\epsilon^{\left(l\right)}\right)\cdot h_{v}^{\left(l-1\right)}+\underset{u\in N(v)}{\sum }h_{u}^{\left(l-1\right)}\right) & & \end{array}$$ (7)

where $h_{v}^{\left(l\right)}$ represents the feature vector of node *v* at layer *l*; $\epsilon^{\left(l\right)}$ is a learnable parameter used to adjust the importance of the node’s own features; *N* ( *v* )  denotes the set of neighboring nodes of node *v*; and $h_{u}^{\left(l-1\right)}$ is the feature vector of node *u* at layer *l* − 1.

Similar to the aggregation process mentioned above, K-hop Conv extends this foundation. Specifically, the K-hop feature representation of node at layer is given by:


$$\begin{array}{ccc} H_{v}^{\left(l,0\right)}=h_{v}^{\left(l-1\right)} & & \end{array}$$ (8)

The initial *K*-hop value of node *v* is set as the node embedding at each layer. By repeating the propagation *k* times, the model can effectively gather information within the *K*-hop distance.


$$\begin{array}{ccc} H_{v}^{\left(l,k\right)} & ={\text{MLP}}^{\left(l,k\right)}\left(\left(1+\epsilon^{\left(l,k\right)}\right)\cdot H_{v}^{\left(l,k-1\right)}+N_{v}^{\left(l,k-1\right)}\right) & \end{array}$$ (9)

$$\begin{array}{ccc} N_{v}^{\left(l,k-1\right)} & =\underset{u\in N(v)}{\sum }H_{u}^{\left(l,k-1\right)} & \end{array}$$ (10)

where *l* denotes the number of layers in the convolutional network, and *k* represents the number of hops in a particular layer. $H_{v}^{\left(l,k\right)}$ denotes the feature of node *v* at layer *l*, step *k*; $\epsilon^{\left(l,k\right)}$ is a learnable parameter at layer *l*, step *k*; $N_{v}^{\left(l,k-1\right)}$ represents the aggregated neighbor features of node *v* at layer *l*, step *k*; and ${\text{MLP}}^{\left(l,k\right)}$ is the multilayer perceptron at layer *l*, step *k*, used for nonlinear transformation.

The aggregation process also takes into account the differences in node degrees, using different scales to manage the aggregation process, which can be represented as:


$$\begin{array}{ccc} h_{v}^{\left(l\right)}=\underset{k}{\sum }s^{\left(l,k,d\right)}H_{v}^{\left(l,k\right)},\;h_{v}^{\left(0\right)}=F & & \end{array}$$ (11)

where $s^{\left(l,k,d\right)}$ controls the scale of *k* features’ weight at layer *l*, where *d* represents the degree of node *v*. The node embedding in layer *l* is updated based on the weighted *K*-hop features, and when *l* = 0, this corresponds to the feature input *F* formed through Feature Engineering.

**Virtual node.** Although the K-hop convolution expands the maximum interaction distance, it still fails to directly capture the relationships between certain long-distance node pairs. To address this issue, Gilmer et al. proposed the GCN-VN variant [44], which enhances the flow of information by introducing a virtual node that connects to all other nodes in the graph. During each round of message passing, every original node in the graph exchanges information with this virtual node. The design of the virtual node module is inspired by GCN-VN, as shown in Fig 2E. The function (a summation operation) aggregates the node embeddings, summing all node embeddings to form the aggregated information of the entire graph, defined as:


$$\begin{array}{ccc} h_{G}^{\left(l\right)}=\text{READOUT}\left(\left\{h_{v}^{\left(l\right)}|v\in V\right\}\right)=\underset{v\in V}{\sum }h_{v}^{\left(l\right)} & & \end{array}$$ (12)

The aggregated node features are then fed into a feedforward network to obtain a global understanding of the entire graph. We refer to this as the virtual node, defined as:


$$\begin{array}{ccc} h_{vn}^{\left(l\right)}=\text{FFN}\left(h_{G}^{\left(l\right)}\right) & & \end{array}$$ (13)

The updated virtual node embedding is then broadcast to each node in the graph to update the node features, defined as:


$$\begin{array}{ccc} h_{v}^{\left(l\right)}={\text{UPDATE}}_{v}^{\left(l\right)}\left(h_{v}^{\left(l-1\right)},\text{Broadcast}\left(h_{vn}^{\left(l-1\right)}\right)\right) & & \end{array}$$ (14)

where $h_{G}^{\left(l\right)}$ represents the global aggregated information of the graph at layer *l*; $h_{vn}^{\left(l\right)}$ represents the virtual node at layer *l*.

**Linear attention.** The K-hop convolution and Virtual Node modules effectively capture both local and global information within the graph data, but they fall short in fully achieving dynamic adjustment of global graph information [45]. Therefore, the final component of TGF-M’s predictor is the introduction of an attention mechanism, which directly adjusts the weights and aggregates information from all nodes across the entire graph, ensuring the flexible capture of molecular graph information. Another significant advantage of the linear attention method used here is that it reduces the computational complexity from $O(n^{2})$ to *O* ( *n* )  through a specific linearization technique, which significantly reduces computational complexity, making it highly suitable for large-scale graph data. As shown in Fig 2F, TGF-M employs a Linear Transformer to implement the attention mechanism, defined as follows:


$$\begin{array}{ccc} \begin{array}{ccc} Q^{\left(l\right)} & ={\text{FFN}}_{Q}\left(h_{v}^{\left(l\right)}\right), & \\ K^{\left(l\right)} & ={\text{FFN}}_{K}\left(h_{v}^{\left(l\right)}\right),\\ V^{\left(l\right)} & ={\text{FFN}}_{V}\left(h_{v}^{\left(l\right)}\right) \end{array} & & \end{array}$$ (15)

Next, the key vector *K* is multiplied by the value vector *V*. This step simplifies the computation between the query vector *Q* and the key-value pairs, similar to the concept of the Kernel Trick [46], thereby reducing computational complexity. The Readout function is then used to aggregate the *KV* matrix, integrating multiple weighted results. The query vector *Q* performs a dot product with the output from the *READOUT* layer, using *Q* to focus on the processed information from *K* and *V*, forming the final output, defined as:


$$\begin{array}{ccc} h_{v}^{\left(l+1\right)}=Q^{\left(l\right)}\cdot \text{Readout}\left(K^{\left(l\right)}\times V^{\left(l\right)}\right) & & \end{array}$$ (16)

where *Q*, *K*, and *V* represent the query, key, and value vector matrices, respectively.

Next, these three key components are utilized through a multi-layer nested loop, allowing the predictor to fully leverage the features encoded by feature engineering. Ultimately, this process enables the accurate prediction of the molecular HOMO-LUMO gap through Readout, Normalization, and a Feedforward Neural Network (FNN).

**Table 1:** The benchmark model of the PCQM4MV2.

Model	Model Type	# Param	Complexity	MAE*↓*
GCN	GNN	2.0M	*O* ( *n* + *m* )	0.1379
GAT	GNN	6.7M	*O* ( *n* + *m* )	0.1302
GIN	GNN	3.8M	*O* ( *n* + *m* )	0.1195
GAT-VN	GNN	6.7M	*O* ( *n* + *m* )	0.1192
GCN-VN	GNN	4.9M	*O* ( *n* + *m* )	0.1153
GIN-VN	GNN	6.7M	*O* ( *n* + *m* )	0.1083
CoAtGIN	GNN	6.4M	*O* ( *n* + *m* )	0.0901
TokenGT	Transformer	48.5M	$O({\left(n+m\right)}^{2})$	0.0910
GRPE	Transformer	46.2M	$O(n^{2})$	0.0890
EGT	Transformer	89.3M	$O(n^{2})$	0.0869
Graphormer	Transformer	48.3M	$O(n^{2})$	0.0864
GPS++	Transformer+MPNN	44.3M	$O(n^{2})$	0.0778
Transformer-M	Transformer	68M	$O(n^{2})$	0.0772
Uni-Mol+	Transformer	77.0M	$O(n^{3})$	0.0693
TGT-At	Transformer	203.9M	$O(n^{3})$	0.0671
TGF-M	GNN	6.4M	*O* ( *n* + *m* )	0.0647

## Results and discussion

### Performance of the TGF-M and benchmark

The benchmark results shown in Table 1 reflect the performance on the PCQM4Mv2 dataset. Graph Neural Networks (GNNs) prioritize local graph structure, offering computational efficiency but struggling with long-range dependencies. In contrast, Transformers excel at modeling global relationships, providing higher accuracy but at the expense of increased computational complexity due to the self-attention mechanism. The results clearly indicate that there is an inevitable trade-off between model prediction accuracy and computational complexity, with higher prediction accuracy often accompanied by a significant increase in computational complexity. Traditional GNNs models such as GCN [26], GAT [47] , GIN [27], and their variants (GCN-VN, GAT-VN, GIN-VN) have relatively small parameter sizes and are known for their low computational complexity. However, in terms of prediction accuracy, their MAE values range from 0.1083 to 0.1379, falling short of achieving optimal prediction performance. Lightweight models like CoAtGIN [30] demonstrate relatively high prediction accuracy while maintaining a low number of parameters. Large-scale parameter models such as TokenGT [48], GRPE [32], EGT [34], Graphormer [31], GPS++ [35], and Transformer-M [36] excel in prediction accuracy due to their more complex model structures. However, the significantly increased computational cost of these models may limit their applicability in resource-constrained environments.

In our experiments, TGF-M’s hyperparameters were set to a batch size of 512 and an embedding dimension of 256. With just 100 epochs of training on the re-segmented PCQM4Mv2, the model achieved an impressive MAE of 0.0647 using only 6.4M parameters. This demonstrates that the TGF-M model excels in both prediction accuracy and computational complexity .

### Comparison with state-of-the-art models

To eliminate the potential impact of re-segmenting the dataset and achieve a direct comparison of model performance, we randomly selected 1,000 molecules from the official validation set of PCQM4Mv2, defining it as a unified validation interval. We supplemented the missing 3D information in the validation set by obtaining the SDF data of these molecules from the PubChem website. Based on this improved dataset, we conducted an in-depth comparison of the trained TGF-M model with the two latest SOTA models on the OGB official leaderboard—TGT-At [39] and Uni-Mol+ [38]. We performed a comprehensive analysis and presentation of the experimental results.

**Fig 3:**
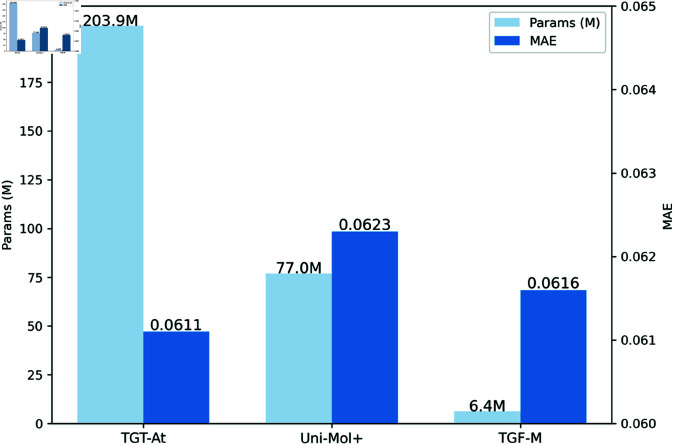
Bar chart of model’s parameters and MAE. Light blue bars indicate the parameter count, while dark blue bars indicate the MAE metric.

All three models leverage molecular 3D information. TGT-At and Uni-Mol+ employ modified Transformer architectures with a time complexity of $O(n^{3})$. In contrast, TGF-M adopts a novel topology-augmented geometric feature encoder, achieving results comparable to large-parameter models using only a traditional GNN-based predictor, with a time complexity of just *O* ( *n*  +  *m* ) . As shown in the experimental results in Fig 3, the TGF-M model achieved an MAE of 0.0616, slightly higher than the 0.0611 of TGT-At but better than the 0.0623 of Uni-Mol+. Although TGF-M slightly underperforms TGT-At in terms of MAE, the difference is minimal, demonstrating TGF-M’s strong competitiveness in prediction accuracy, especially considering its parameter count and training cost. These findings indicate that TGF-M is highly suitable for large-scale molecular modeling and practical applications in computational biology.

**Fig 4:**
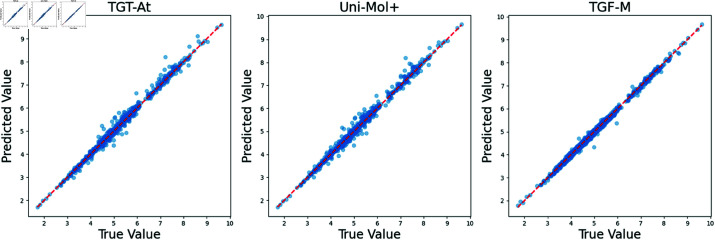
Comparison of scatter plots between predicted and true values across models. The horizontal axis denotes the true value, while the vertical axis denotes the predicted value. The closer the sample points are to the diagonal red line, the better the predictive performance.

Analysis of the scatter plot in Fig 4 shows that the TGF-M model achieves a very high degree of fit between the predicted and actual values, with scatter points evenly distributed along the ideal diagonal line, indicating that the model has strong generalization capabilities. Compared to the TGT-At and Uni-Mol+ models, TGF-M model maintains a high level of consistency in prediction accuracy with fewer outliers, demonstrating its reliability for high-precision molecular property predictions. These results highlight TGF-M’s potential for practical applications in chemical property prediction and molecular design, where accuracy and stability are critical.

**Fig 5:**
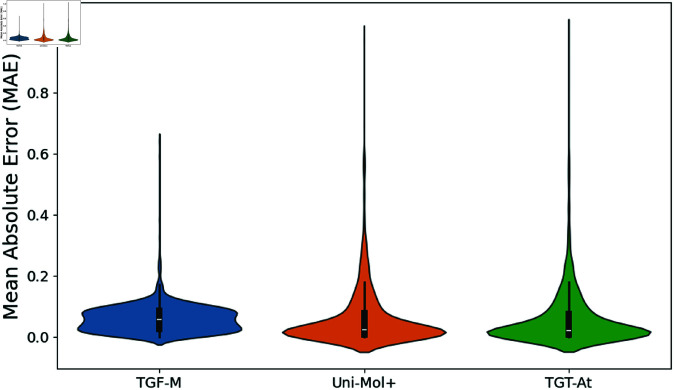
Violin plot comparison of the predictions from models. Thicker sections represent areas of higher data density, while thinner sections indicate fewer observations. The central marker typically shows the median.

Fig 5 presents the distribution of the mean absolute error (MAE) for the three models. The violin plot reveals that the error distribution of the TGF-M model is more concentrated, with a lower median, indicating that this model performs the most stably on the test dataset. In contrast, although Uni-Mol+ has a lower median, its distribution shows a noticeable long tail, suggesting that it may exhibit higher errors in certain cases, indicating greater variability. The TGT-At’s performance falls between the two, with a slightly more dispersed error distribution, but overall it also shows relatively low errors. This further validates that the TGF-M model, while ensuring accuracy, can effectively control prediction errors, making the prediction results more stable and reliable.

### Discussion on additional topological information

To investigate how topological information enhances geometric representations, we conducted supplementary experiments within the TGF-M framework, using two strategies: with edge attributes and use feature fusion. This approach aims to better understand how different types of topological information enhance geometric information.

**With edge attributes.** In the edge feature addition component, we analyzed the bond types, stereochemistry, and conjugation in the 2D molecule information, extracting 11 key topological features. These features were then scaled and offset through an embedding layer during the Gaussian encoding process to enable specific recognition and processing of different types of topological edges. The specific method is defined as follows:


$$\begin{array}{ccc} D^{\prime }=\partial^{\left(\tau \right)}\cdot D+\beta^{\left(\tau \right)} & & \end{array}$$ (17)

where *D* represents the set of interatomic distances for all edges in the 3D molecule; $D^{\prime }$ is the set of 3D molecular geometric distances embedded with chemical bond topological features; $\partial^{\left(\tau \right)}$ and $\beta^{\left(\tau \right)}$ are the scaling and offset parameters for different edge types, determined by the topological features *τ* of the chemical bonds.

**Use feature fusion.** In the feature fusion part, we utilized learnable attention mechanism parameters to dynamically fuse the structural encodings of 2D and 3D molecules. The attention mechanism assigns weights to different features, emphasizing or de-emphasizing specific information during the fusion process. This method allows us to dynamically observe the roles of 2D and 3D molecular representations in downstream tasks during experiments, providing a better understanding of their contributions to task performance.

**Table 2:** Experimental results of adding different topological information.

Model	Param	MAE*↓*
TGF-M	6,391,041	0.0647
With edge attributes	6,391,063	0.0675
Use feature fusion	6,394,882	0.0790

**Fig 6:**
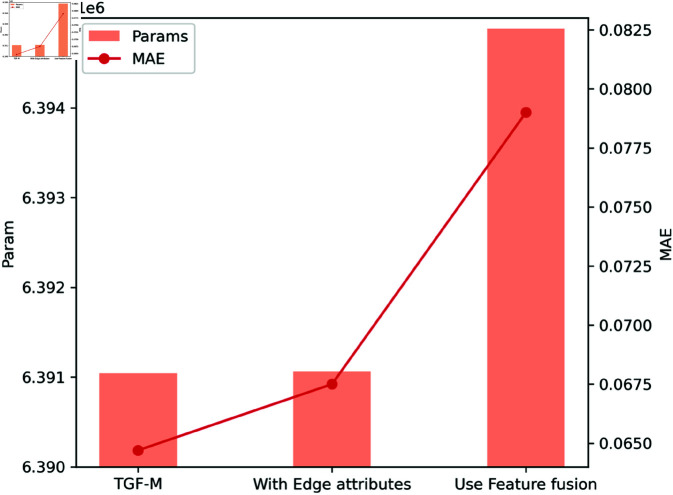
Line chart and Bar chart of models’ parameters and MAE. The line chart represents the parameter values, while the bar chart illustrates the MAE.

The experimental results from Table 2 show that any additional topological information introduced on top of the TGF-M’s topology-enhanced geometric strategy actually leads to a decrease in model performance. This suggests that TGF-M’s topology-enhanced geometric feature method is already sufficiently comprehensive and advanced, with its enhancement of geometric distance information by topological connectivity and degree information being adequate to meet the training requirements of the PCQM4Mv2 dataset. Fig 6 clearly shows the trend of changes in model parameters and MAE before and after adding different topological information.

### Ablation study

To investigate the contribution of each module in TGF-M, we conducted an ablation study using the controlled variable method, which isolates the effect of individual components by systematically removing or modifying them in the model.

We examined the impact of different molecular encoding features in the Feature Engineering component, specifically including: single atomic features without bond information (Atom), 2D topological information only (Topology), 3D geometric information only (Geometric), and the combination of topology-enhanced geometric information (TGF-M). As shown in the results in Table 3, topology-enhanced geometric information achieved the best model performance, demonstrating that combining geometric and topological information is crucial. This indicates that topological information significantly enhances the representation power of geometric features, which is critical for more accurate molecular modeling.

**Table 3:** Ablation study results on different molecular encoding features.

Module				Model	Param	MAE*↓*
Gauss Encoding	Edge Scatter	Degree Scaler	Atom feature			
✗	✗	✗	✓	Atom	6,389,505	0.0891
✗	✓	✓	✓	Topology	6,393,857	0.0882
✓	✓	✗	✓	Geometric	6,390,017	0.0688
✓	✓	✓	✓	TGF-M	6,391,041	0.0647

Furthermore, we evaluated the impact of the K-hop convolution, Virtual Node, and Linear Attention components in the Predictor. The results in Table 4 show that as the number of convolution layers increased from 1-hop to 3-hop, model performance progressively improved, with the best performance achieved at 3-hop convolution (TGF-M). This demonstrates that increasing the convolutional receptive field enables the model to capture more complex and long-range relationships within molecular structures, thereby improving the model’s prediction accuracy. Additionally, the results suggest that the Virtual Node and Linear Attention modules play critical roles in enhancing model performance. These components work synergistically, with each module complementing the others, leading to better feature aggregation and improving overall model performance.

**Table 4:** Ablation study results on predictor modules.

Model	Param	MAE*↓*
1-hop Conv	4,089,089	0.0687
2-hop Conv	5,240,065	0.0669
3-hop Conv (TGF-M)	6,391,041	0.0647
Without Virtual Node	5,536,001	0.0678
Without Linear Attention	5,503,233	0.0656

### Exploration of chemical interpretation

#### T-SNE visualization of model outputs.

**Fig 7:**
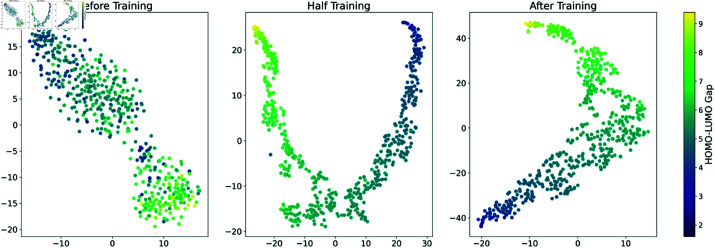
T-SNE visualization at different stages of the training process. The color intensity denotes the magnitude of the molecular energy gap, and the training process organizes the molecular representations within the latent space in a structured manner.

To comprehensively validate the performance and interpretability of the TGF-M model on the PCQM4Mv2 dataset, we employed T-SNE to visualize molecular representations across a particular of batch. In the visualizations shown in Fig 7, each dot represents a molecule in a two-dimensional plane, colors represent the specific HOMO-LUMO gap values for each molecule. After training the TGF-M model, the molecular representations in latent space became considerably ordered, with molecules having similar HOMO-LUMO gaps clustering together to form a distinct gradient. This indicates that the TGF-M model successfully captured and enhanced key physicochemical features associated with the HOMO-LUMO gap, leading to a closer alignment of molecular representations in latent space for molecules with similar gaps.

#### Interpretability of the learning process.

Additionally, the visualization results at different training stages shown in Fig 7 reveal that as the model training progresses, the data distribution in the T-SNE visualization gradually shifts from a disordered state to an ordered pattern.

Before the training, the data points in the T-SNE space are scattered and lack obvious structure, indicating that the model has not yet captured the relationship between molecular features and the energy gap. Half the training, the model has partially captured key molecular features, enabling it to cluster molecules with similar energy gaps together. However, the data distribution remains unclear, the color gradient changes are inconsistent, and the clusters are not tight, particularly at the ends of the “U” shape, indicating shortcomings in the model’s feature learning. After the training, the model has fully captured the relationship between molecular features and the energy gap, resulting in a clear and ordered data distribution in the T-SNE space, demonstrating the model’s deep understanding and predictive capability of molecular structures.

This evolutionary process illustrates how the model gradually learns the intrinsic relationship between molecular structure and the energy gap, validating its predictive capability and revealing how it internalizes and represents these chemical properties during the learning process.

#### Relationship between molecular structure and energy gap.

From a chemical perspective, conjugation reduces the HOMO-LUMO energy gap by increasing *π*-electron delocalization, thereby lowering the energy difference within the molecule and influencing its optical and electrical properties. Aromaticity, on the other hand, stabilizes the HOMO-LUMO gap further through highly symmetric *π*-electron delocalization, typically resulting in a smaller HOMO-LUMO gap and enhanced molecular stability [49].

To ensure that the model provides chemically interpretable results, we classified the molecules based on two key chemical properties that influence the HOMO-LUMO gap: conjugation and aromaticity. We then visualized the distribution of molecular representations from a particular batch after training using T-SNE in the reduced-dimensional space. The data was projected into three dimensions, where the x and y axes represent the two dimensions of T-SNE, and the third axis indicates the size of the HOMO-LUMO gap. Additionally, a two-dimensional space omitting the HOMO-LUMO gap was provided to better illustrate the classification results. Red and blue points represent conjugated and non-conjugated molecules, while purple and yellow points represent aromatic and non-aromatic molecules.

**Fig 8:**
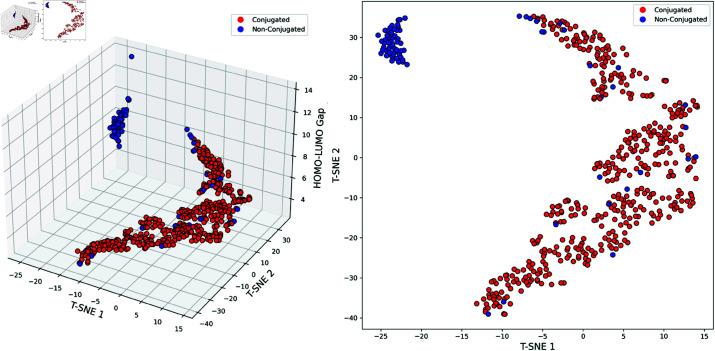
T-SNE visualization of Conjugated vs. Non-Conjugated molecules. Red indicates conjugated molecules, while blue indicates non-conjugated ones. The results illustrate the projection of various molecules in two-dimensional and three-dimensional space after model training.

**Conjugation. **The T-SNE visualization in Fig 8 shows a significant separation between conjugated and non-conjugated molecules in the reduced-dimensional space. Conjugated molecules cluster in regions with smaller HOMO-LUMO gaps, while non-conjugated molecules are more dispersed, tending towards larger gaps. The *π*-electron delocalization in conjugated molecules results in smaller and more concentrated HOMO-LUMO gaps, whereas non-conjugated molecules, lacking this effect, exhibit larger and more diverse gaps.

**Fig 9:**
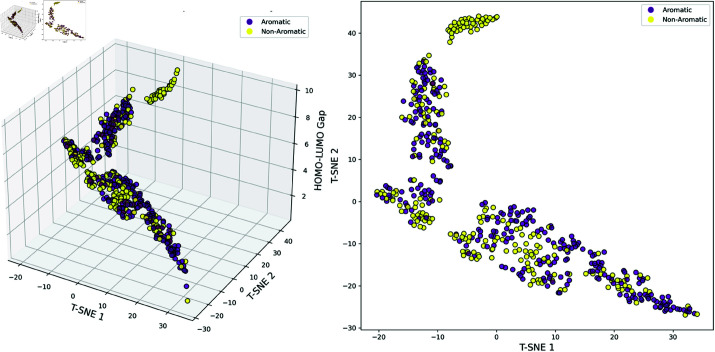
T-SNE visualization of Aromatic vs. Non-Aromatic molecules. Purple indicates Aromatic molecules, while yellow indicates Non-Aromatic molecules. The results illustrate the projection of various molecules in two-dimensional and three-dimensional space after model training.

**Aromaticity. **The T-SNE visualization in Fig 9 shows a clear separation between aromatic and non-aromatic molecules. Aromatic molecules are concentrated in specific regions, while non-aromatic molecules are more widely dispersed. The three-dimensional plot further highlights the strong correlation between aromaticity and the HOMO-LUMO gap. Due to their stable *π*-electron systems, aromatic molecules exhibit smaller and more clustered HOMO-LUMO gaps, displaying a tendency to aggregate. In contrast, non-aromatic molecules, lacking this stability, have more widely distributed and dispersed HOMO-LUMO gaps.

The results indicate that the model effectively distinguishes molecular structural features. Additionally, the relationship between molecular structure and energy gap revealed in the three-dimensional plot aligns with established theories in the field, providing chemically interpretable support for the model.

## Conclusion

In this study, we addressed the challenge of balancing prediction accuracy with computational complexity in molecular property prediction. We introduced TGF-M, a novel topology-augmented geometric feature encoder that effectively integrates molecular topological and geometric information. This approach not only enhances predictive accuracy but also reduced computational complexity.

To validate the proposed method, we conducted extensive experiments. Comparative benchmarks confirmed TGF-M’s strong generalization capabilities, achieving competitive accuracy with minimal computational resources. Ablation studies further highlighted the critical roles of key components, in achieving optimal performance. Additionally, T-SNE visualizations revealed the chemical interpretability of TGF-M, confirming its ability to capture meaningful structural and energetic patterns consistent with established chemical principles. These results validate TGF-M as a robust and efficient model, suitable for large-scale molecular modeling tasks and practical applications in computational biology, particularly in resource-constrained environments.

While TGF-M effectively balances predictive accuracy and computational efficiency, one notable drawback is its reliance on 3D structural data, which can be computationally expensive to obtain and process. In future work, we will focus on predicting interatomic geometric distances directly from 2D molecular graphs, thereby removing the dependence on 3D data. This advancement is expected to reduce computational costs, enable large-scale applications, and enhance accessibility for tasks such as drug discovery and material screening—all while leveraging inherent structural connectivity to provide interpretable predictions.
